# Mucosal Heterologous Prime/Boost Vaccination Induces Polyfunctional Systemic Immunity, Improving Protection Against *Trypanosoma cruzi*

**DOI:** 10.3389/fimmu.2020.00128

**Published:** 2020-02-21

**Authors:** Andrés Sanchez Alberti, Augusto E. Bivona, Marina N. Matos, Natacha Cerny, Kai Schulze, Sebastian Weißmann, Thomas Ebensen, Germán González, Celina Morales, Alejandro C. Cardoso, Silvia I. Cazorla, Carlos A. Guzmán, Emilio L. Malchiodi

**Affiliations:** ^1^Facultad de Farmacia y Bioquímica, Cátedra de Inmunología and Instituto de Estudios de la Inmunidad Humoral “Prof. Ricardo A. Margni” (IDEHU), UBA-CONICET, Universidad de Buenos Aires, Buenos Aires, Argentina; ^2^Departamento de Microbiología, Parasitología e Inmunología, Facultad de Medicina, Instituto de Microbiología y Parasitología Médica (IMPaM), UBA-CONICET, Universidad de Buenos Aires, Buenos Aires, Argentina; ^3^Department of Vaccinology and Applied Microbiology, Helmholtz Centre for Infection Research, Braunschweig, Germany; ^4^Departamento de Patología, Facultad de Medicina, Instituto de Fisiopatología Cardiovascular, Universidad de Buenos Aires, Buenos Aires, Argentina

**Keywords:** neglected tropical disease, Chagas disease, Anti-Trypanosoma cruzi vaccine, prime-boost vaccine, Traspain, cyclic-di-AMP, T cell polyfunctionality, cell-mediated immunity

## Abstract

There are several unmet needs in modern immunology. Among them, vaccines against parasitic diseases and chronic infections lead. *Trypanosoma cruzi*, the causative agent of Chagas disease, is an excellent example of a silent parasitic invasion that affects millions of people worldwide due to its progression into the symptomatic chronic phase of infection. In search for novel vaccine candidates, we have previously introduced Traspain, an engineered trivalent immunogen that was designed to address some of the known mechanisms of *T. cruzi* immune evasion. Here, we analyzed its performance in different DNA prime/protein boost protocols and characterized the systemic immune response associated with diverse levels of protection. Formulations that include a STING agonist, like c-di-AMP in the boost doses, were able to prime a Th1/Th17 immune response. Moreover, comparison between them showed that vaccines that were able to prime polyfunctional cell-mediated immunity at the CD4 and CD8 compartment enhanced protection levels in the murine model. These findings contribute to a better knowledge of the desired vaccine-elicited immunity against *T. cruzi* and promote the definition of a vaccine correlate of protection against the infection.

## Introduction

Chagas disease is a potentially life-threatening disease caused by the protozoan parasite *Trypanosoma cruzi*. It is recognized by WHO as a neglected tropical disease in Latin America, where more than 70 million people are at risk of contracting the infection (1).

According to last WHO estimates, about 6–7 million people worldwide are infected with *T. cruzi*. Vectorial transmission occurs when a triatomine bug feeds on mammalian blood and defecates over the skin. The feces of the vector contain the parasite and can be introduced through scratching or by mucosa. Prevention measures have historically been focused on domiciliary vectorial control, blood transfusion, and more recently, congenital screening programs (2). The trypanocidal drugs available are highly effective during the acute phase, but treatment of the chronic phase remains an unsolved roadblock (3). About 30% of chronically infected people develop cardiac alterations and up to 10% develop digestive, neurological, or mixed forms, which are responsible for disability and death during the chronic phase (4).

**Figure 1:**
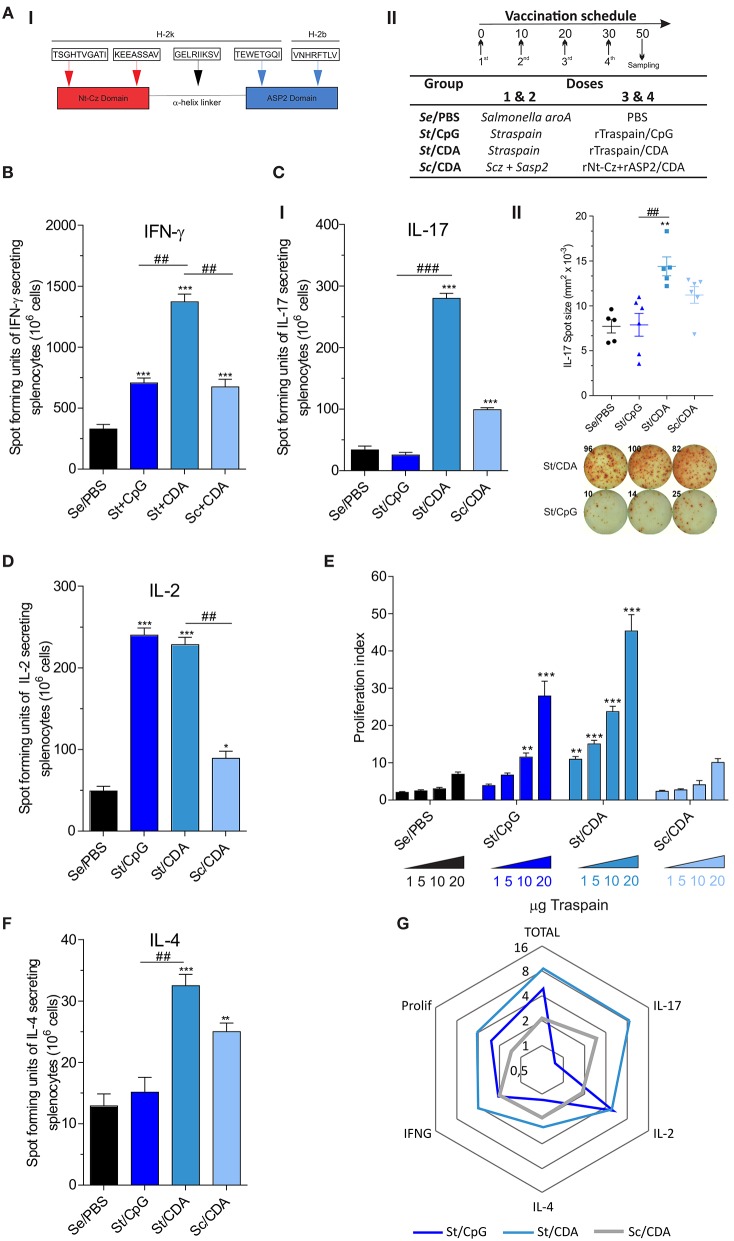
Profile of the immune response triggered by different prime-boost vaccination protocols. **(A)** I—Traspain cartoon showing main domains of the molecule and selected murine MHC-I epitopes that have been shown immunogenic. II—Immunization schedule and vaccine formulation received by each group. Secreted cytokines were determined by ELISPOT assay. Pooled-splenocytes were restimulated with RPMI or Traspain, and mean number of spot-forming units were determined for the indicated cytokine: **(B)** IFN-γ **(C)** I—IL-17, II—IL-17 spot size and representative image showing the response of spleen cell from mice that received Traspain plus CpG or CDA as an adjuvant. **(D)** IL-2 and **(F)** IL-4. *p < 0.05, **p < 0.01, ***p < 0.001 against control group (Se/PBS). ^##^*p* < 0.01, ^###^*p* < 0.001 between the indicated groups, one-way ANOVA + Tukey's multiple comparisons test. **(E)** Dose-response curve of antigen-specific proliferation assay. Two-way ANOVA + Dunnett's multiple comparisons test. **p < 0.01, ***p < 0.001 against the corresponding category from control group (Se/PBS). Results are expressed as Mean ± SEM, *n* = 18 from 6 female C3H mice per group. **(G)** Radial graph showing average fold of change level of each response variable (cytokines, proliferation, and total response as a sum vs. Se/PBS control group) obtained by each vaccine formulation. Logarithmic scale base 2. All results are representative of two independent experiments.

Even though there is no approved vaccine against Chagas disease, several experimental strategies have been exploited for the development of one, including but not limited to live attenuated parasites (5), subunit vaccines (6–8) (proteins or DNA), and recombinant viral vaccines (9). We have previously introduced Traspain, a novel chimeric antigen rationally designed to display B- and T-cell epitopes of key parasitic protein targets: cruzipain (Cz), amastigote surface protein 2 (ASP2), and a selected region of trans-sialidase (Figure 1AI). This immunogen proved to be both immunogenic and protective against *T. cruzi* murine infection in a protein-subunit vaccine model (10).

Considering the complexity of anti-*T. cruzi* immunity, prime-boost protocols have been carried out in order to increase protection (11, 12). These strategies imply vaccination approaches where the administration of one type of vaccine is followed by a second kind, with the aim of triggering a complementary immune response. Our lab has extensive experience with prime-boost protocols employing DNA priming by orally delivering it with a live attenuated microorganism (*Salmonella enterica* serovar Typhimurium *aroA*) plus a protein boost. Thus, a 4-dose-based regimen was previously tested to improve protection (8, 13). This strategy appears to be an interesting approach considering that multiple boosting in the same site of immunization can cause T-cell sequestration, a fact that has been described as leading to T-cell exhaustion and deletion (14). Boosting with a protein subunit-based vaccine implicates the use of adjuvants to increase immunogenicity. Newly approved adjuvants for humans are focused on TLR ligands like MPLA (TLR4) in the HPV vaccine and ODN-CpG (TLR9) in the new HBV vaccine (15). Between them, the efficacy of CpG has been extensively studied in anti-*T. cruzi* vaccines appearing as an acceptable candidate (8, 13, 16, 17).

T-cell responses are essential for eliminating *T. cruzi*-infected cells (18). However, priming cell-mediated immunity (CMI) through the employment of subunit vaccines models is challenging (19). We have recently reported the efficacy of the STING agonist, 3′5′-c-di-AMP (CDA) for priming pathogen-specific immune responses where Th1/Th17 balanced immunity proved to be protective against this protozoan parasite (10, 20).

IL-17 is a highly versatile pro-inflammatory cytokine that was initially associated with immunopathology and autoimmunity. It has not only a key role acting against extracellular bacteria and fungi but also contributes to the control of intracellular pathogens like *Listeria monocytogenes, Chlamydia muridarum*, and the apicomplexan parasites *Toxoplasma gondii* and *Eimeria falciformis* (21). In the context of vaccine-induced immunity, IL-17 has been shown to contribute to protection against other intracellular pathogens such as *Mycobacterium tuberculosis* (22, 23).

Its role in Chagas disease is still under debate. However, there is plenty of data showing the beneficial effect of IL-17 immunity in *T. cruzi* infection in humans and mice. High levels of this cytokine were detected in patients with better cardiac function in the indeterminate form of the disease (24, 25) or after benznidazole treatment (26). Besides, many experimental studies in mice have found a protective effect of IL-17 by inhibiting an otherwise exaggerated proinflammatory response (27), controlling myocarditis (28), promoting CD8 T-cell priming (29), and even showing more protection than Th1 cells (30). These reports sustain the development of strategies able to prime this type of vaccine-mediated immunity as an effort to improve protection.

Similar to other infections (31), for Chagas disease correlates of vaccine-induced protection remain elusive. Here, we employed Traspain or its components for vaccine formulation in prime-boost protocols and analyzed in detail the systemic immune response triggered by vaccination conferring diverse protection levels in order to better understand the immune response associated with protection.

## Materials and Methods

### Mice and Parasites

Female C3H/HeN (H-2k) mice 6 to 8-weeks-old (Harlan, Rossdorf, Germany) were kept at the animal facility of the Helmholtz Center for Infection Research under specific pathogen-free (SPF) conditions. For challenge studies, mice (Instituto de Microbiología y Parasitología Médica, IMPaM, UBA-CONICET) were kept in the animal facilities of IMPaM. Animal experiments were approved by an ethical board and conducted in accordance to the regulations of Lower Saxony No. 09.42502 04 105/07, Germany, and by the Review Board of Ethics of the School of Medicine, UBA, Argentina (Resol. C.D. # 3721/2014) following the guidelines established by the National Research Council (32). Animal sample size was estimated by a power-based method (33).

For lethal assays, the highly virulent pantropic/reticulotropic RA strain of *T. cruzi* was employed. For the chronic phase analysis, a low virulence myotropic clone was employed (K-98) (34). *T. cruzi* bloodstream trypomastigotes of the RA strain from a discrete typing unit (DTU), VI or K-98 clone (DTU I), were isolated from infected mice and used for challenge studies.

### Immunizations and Challenge

Male or female mice were vaccinated with 4 doses of the prime-boost protocol consisting of oral DNA-prime followed by intranasal protein-boost every 10 days (Figure 1AII), as follows: **Se/PBS**: 2 doses of 10^9^ CFU of *Salmonella enterica* serovar Typhimurium *aroA 7207* (SaroA) carrying empty plasmid pcDNA3.1 orally delivered plus 2 doses of PBS. **St/CpG**: 2 doses of SaroA carrying pcDNA3.1-traspain (St), plus 2 boosts of Traspain + CpG. **St/CDA**: 2 doses of St plus 2 boosts of Traspain+CDA. **Sc/CDA**: 2 doses combining SaroA carrying pcDNA3.1-cruzipain and SaroA carrying pcDNA3.1-asp-2, followed by 2 doses of Nt-Cz + ASP2 + CDA. For the protein boost, groups received 10 μg of each vaccine component, except for Sc/CDA group that received equal molar amounts of each antigen. For lethal challenge assays, 15–30 days after the last dose, mice were infected with 10^3^ *T. cruzi* RA strain blood trypomastigotes by the intraperitoneal route. For sub-lethal assays, 3.10^5^ K98 blood trypomastigotes were administered by the same route.

### ELISPOT Assays

Spleen cells (4 × 10^5^/2 × 10^5^ cells/well) were incubated for 24 h (IFN-γ) or 48 h (IL-2, IL-17, and IL-4) at 37°C with 5% CO^2^, in the absence or presence of 10 μg/ml of Traspain. After incubation, cells were removed, and plates were processed according to the manufacturer's instructions. Colored spots were counted with an ELISPOT reader (CTL S5 Micro Analyzer) and analyzed using ImmunoSpot image analyzer software v3.2 (CTL Europe GmbH, Germany).

### Proliferation Assays

Spleen cells (5 × 10^5^ cells/well) from vaccinated animals were incubated in quadruplicates for 96 h in the presence of different concentrations of Traspain (1, 5, 10, and 20 μg/ml) or the indicated stimulus and proceeded as reported (35). Results were expressed as a proliferation index (PI), calculated as the ratio of mean values from stimulated and RPMI samples.

### Intracellular Cytokine Staining

Splenocytes were isolated and stimulated overnight with 10 μg/ml of Traspain or 10 μM of TEWETGQI peptide in the presence of anti-CD154 PE and anti-CD107 PE-Cy7. Brefeldin A plus monensin were added to cultures during the last 12 h of incubation. Dead cells were stained with LIVE/DEAD^TM^ Fixable Blue Dead Cell Stain Kit (Life Technologies). Surface staining was performed with anti-CD3e V500, anti-CD4-APC-H7 (BD), and anti-CD8α-Brilliant Violet 650 (BioLegend). Cells were fixed at RT with PFA 2%, permeabilized in 0.5% saponin and stained using anti-IFN-γ Brilliant Violet 711 (BioLegend) and anti-TNF-α eFluor450 (eBioscience) in accordance with the manufacturer's instructions.

### Analysis of Polyfunctional Cells

Polyfunctional cells are defined as cells with the ability to produce more than one function at the same time (cytokines and upregulation of activation or degranulation makers, CD154 and CD107a, respectively). Frequencies of each defined subset were determined after automatic Boolean combination gates were employed using FlowJo software. Integrated mean fluorescence intensity (iMFI) was determined by multiplying the MFI of the corresponding channel by the frequency of each subpopulation.

### MHC Class I Multimer Staining

To detect antigen-specific T cells, spleen or blood cells were first labeled with the H2Kk-TEWETGQI dextramer-APC (Immudex) and then with anti-CD3e V500, anti-CD4-APC-H7 (BD), and anti-CD8α-Brilliant Violet V650 (BioLegend) according to the manufacturer's instructions.

### *In vivo* Cytotoxicity Assay

Splenocytes collected from naïve C3H/HeN mice were incubated with 5 μM of the CD8 peptide TEWETGQI or RPMI for 30 min at 37°C and 30 min at 4°C, washed, and then labeled with 10 and 0.5 μM of CFSE (CellTrace™ CFSE Cell Proliferation Kit), respectively. Cells were washed, equally combined, and transferred (4 × 10^7^ total cells) intravenously to syngeneic naïve, immunized, and *T. cruzi*-RA-infected mice at 45 days post-infection (dpi). Spleens were harvested 16 h after transfer, and different CFSE-stained populations were detected by flow-cytometry.

### Assessment of Vaccine Efficacy

Parasitemia and weight loss were monitored every 2 days as previously described by counting peripheral parasites (13). Survival was recorded daily.

Muscle injury was evaluated through the determination of a panel of myopathy-linked enzyme markers at 240 dpi. The assays were performed as previously described (36). The histological features of heart and skeletal (quadriceps) muscles from vaccinated and infected mice were also investigated. A blind histological test was performed as previously described (37). Briefly, fixed material was embedded in paraffin, then sectioned and stained with hematoxylin and eosin. Inflammation was qualitatively evaluated according to the number and spreading of inflammatory foci. Samples were classified with the following score: (1) isolated foci; (2) multiple non-confluent foci; (3) multiple confluent foci; and (4) multiple diffuse foci (38, 39).

#### Electrocardiograms (ECG)

Mice were anesthetized (100 mg ketamine and 16 mg xylazine/kg mouse) at 120 dpi and heart electrical activity was recorded with a Temis TM-300-V electrocardiograph as previously reported (6). Corrected QT interval was calculated by the Bazett formula adapted for mouse (40).

#### Quantitative PCR (qPCR)

Parasite burden in skeletal and heart muscle at 240 dpi was determined by a qPCR adapted from Cummings et al. (41) as previously described (6). Parasite burden was expressed as parasite equivalent/50 ng of total DNA.

### Statistical Analysis

Statistical analysis was carried out with GraphPad Prism 6.0 software (San Diego, CA, USA) using one-way or two-way ANOVA. The number of animals, specified in figure legends, was estimated by a power analysis comparing the size of the difference in the variable of interest between immunized and control groups based on either previous or pilot experiments. *p* < 0.05 were considered significant. Homoscedasticity was tested employing Levene's test in all ANOVA. Normality was checked using the Shapiro-Wilk test and/or quantile-quantile plot, QQPLOT using R software (42).

## Results

### Profiles of the Immune Response Obtained Upon Different Prime-Boost Strategies

In order to evaluate the influence of the adjuvant and antigen in prime-boost vaccines, we analyzed the frequency of antigen-specific cytokine secreting cells in splenocytes by an ELISPOT assay (Figure 1). The frequency of IFN-γ secreting cells was higher in vaccinated groups compared to Se/PBS control group (Figure 1B). The highest frequency of these cells was detected in St/CDA group, which presented nearly a two-fold increment compared to other immunized groups. Interestingly, a marked difference was detected in the numbers of vaccine-specific IL-17 secreting cells, where only groups that received the CDA boost in the formulation were able to increase its frequency compared to control animals (Figure 1CI). Moreover, not only was the frequency different but also a significant contrast in the size and intensity of each spot-forming unit (SFU) was detected (Figure 1CII). This points out to the fact that CDA vaccinated mice have a higher ability to secrete this cytokine. On the contrary, no difference was observed in the levels of IL-2 secreting cells between CpG and CDA groups (Figure 1D). Notwithstanding, the group that received Sc/CDA showed a lower frequency of SFU in the majority of cytokines analyzed. A similar trend was observed in the proliferative ability of spleen cells upon antigen re-encounter where Sc/CDA group showed the worst performance (Figure 1E). All immunized mice displayed low levels of IL-4 secreting cells, being the ratio IFN-γ/IL-4 ≈ 46, 40, 27 for St/CpG, St/CDA, and Sc/CDA, respectively. These results highlight the bias toward a Th1 profile in the adjuvants employed. A fold of change analysis of each variable reflected the increase in vaccine potency observed in St-groups as opposed to antigen combination. Remarkably, CDA as a boost adjuvant displayed a more balanced cytokine profile with higher presence of IL-17 and IL-4 than CpG (Figure 1G). This broader Th profile might help to avoid pathology and contribute to a better control of *T. cruzi* infection.

### Vaccine Efficacy During the Acute Phase of *T. cruzi* Infection

**Figure 2:**
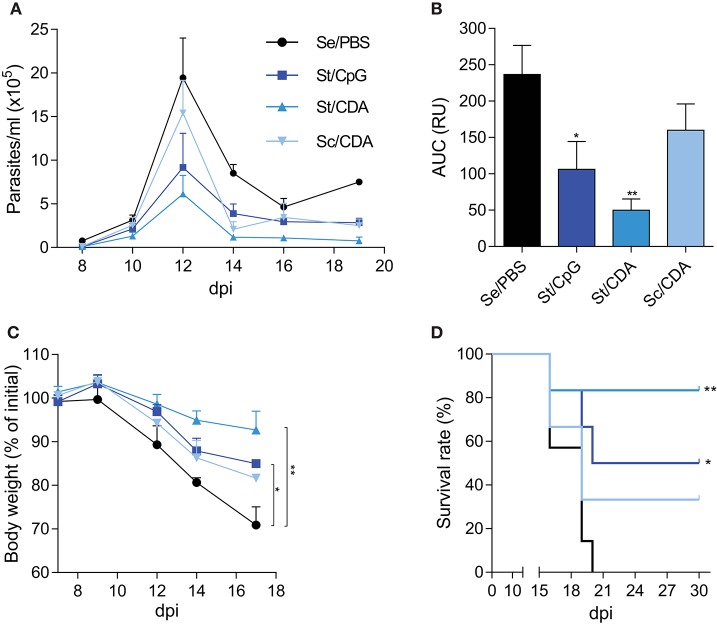
Improved efficacy in St/CDA vaccinated mice upon a lethal challenge with *T. cruzi* RA strain. Female C3H mice were vaccinated and 15–30 days after last dose were intraperitoneally infected with 1,000 blood trypomastigotes of *T. cruzi* RA strain **(A)** Parasitemia. **(B)** Area under the curve (AUC) of parasitemia up to 14 dpi. RU, relative units **p < 0.01, *p < 0.05 differences were calculated with respect to Se/PBS control group, one-way ANOVA + Bonferroni post-hoc test. **(C)** Weight loss. Results are expressed as percentage with respect to pre-infection **p < 0.01, *p < 0.05 at 17 dpi between the indicated groups, one-way ANOVA + Dunnett post-test. **(D)** Survival rate. Asterisks indicate significant difference with respect to Se/PBS control group. **p < 0.01, *p < 0.05 Log-rank test. *n* = 5–7 mice per group. Results are representative of three independent experiments.

In order to analyze the protection conferred by each formulation, immunized female C3H mice were challenged with a lethal dose of the highly virulent RA strain of *T. cruzi*. This model of infection is well-established in our lab (6, 16, 36, 43). The number of blood trypomastigotes, weight loss, and the survival rate were employed as endpoints for assessing vaccine performance. Due to the higher sensibility in the analyzed readouts, this infection model is ideal to determine protection in the acute phase of infection. All vaccinated groups showed lower parasitemia than control animals (Figure 2A). However, mice immunized with St/CDA showed nearly a five-fold reduction in the number of circulating parasites along the acute phase compared to control animals (Figure 2B); area under the curve values: AUC_Se/PBS_ = 237 vs. AUC_St/CDA_ = 49.8 (p = 0.0038).

Weight loss, though detected in all mice, was ameliorated in vaccinated groups compared with control animals (Figure 2C). Thus, at 17 dpi, control mice showed the highest loss (nearly 30% of their initial mass), this difference being significant compared to Traspain-vaccinated animals. This result denotes the improvement on disease severity when animals received Traspain as vaccine. In that way, the reduced parasitemia and weight loss of St/CDA group were associated with an increase in survival rate among immunized animals (Figure 2D). Conversely, Sc/CDA group showed the worst outcome in terms of parasitemia (AUC_Sc/CDA_: 160, *p* = 0.32 vs. Se/PBS), weight loss (*p* = 0.19 at 17 dpi), and survival (*p* = 0.45 vs. Se/PBS) compatible with a scenario of higher disease severity upon infection.

### Heterotypic Protection Against a Sub-Lethal *T. cruzi* Challenge

To analyze the protective capacity of each prime-boost strategy against a *T. cruzi* strain with a different outcome, a non-lethal model of experimental infection was established combining *T. cruzi* K-98 clone (DTU I) with male C3H mice based on their higher susceptibility to infection (44, 45) and on previous reports of similar infection models (31, 46). Mice were vaccinated and subsequently infected with K-98 blood trypomastigotes.

**Figure 3:**
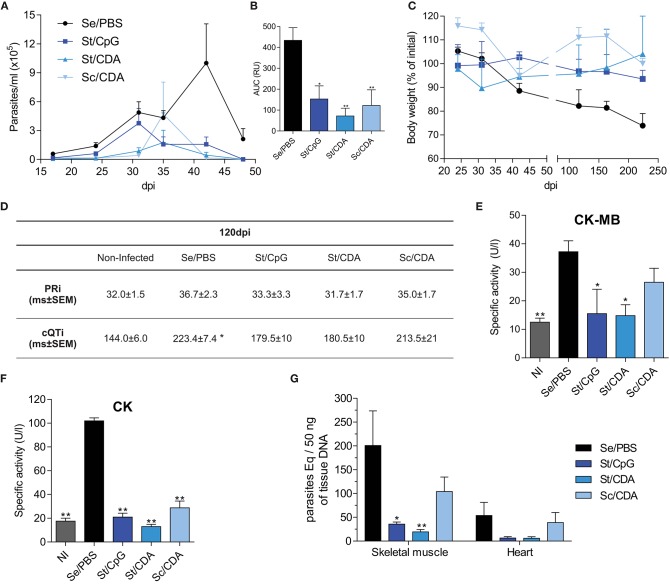
Assessment of vaccine efficacy during parasitic chronic infection with a vaccine unrelated strain. Male C3H mice were vaccinated and 30 days after last dose were infected with blood trypomastigotes of *T. cruzi* K-98 strain. **(A)** Parasitemia. **(B)** Area under the curve (AUC) of parasitemia. RU, relative units **p < 0.01, *p < 0.05 differences were calculated with respect to the Se/PBS control group, one-way ANOVA + Bonferroni post-hoc test. **(C)** Weight loss. Results are expressed as percentage with respect to pre-infection. **(D)** Electrocardiogram parameters: corrected QT interval (cQTi) and PR interval (PRi), *p < 0.05 against non-infected mice, one-way ANOVA + Dunn's post-test. Serum activity of cardiomyopathy-associated enzymes. **(E)** Creatine kinase MB isoform (CK-MB). **(F)** Creatine kinase (CK), from immunized infected mice. NI, non-infected. *p < 0.05, **p < 0.01 compared to Se/PBS group, bars indicate significant differences, *p* < 0.05, between the indicated groups, one-way ANOVA + Dunnett's post-test. **(G)** Parasite load by qPCR in target tissues, *p < 0.05, **p < 0.01 compared to Se/PBS group, two-way ANOVA + Dunnett's post-test. *n* = 3–7 mice per group. All results are representative of at least two independent experiments.

As Figures 3A,B shows, vaccinated animals were able to significantly control parasitemia during the acute phase compared to Se/PBS control mice. In that way, all of them were able to efficiently reduce the highest peak of parasites at 42 dpi. Albeit, St/CpG and Sc/CDA groups presented higher peaks earlier, around 30–40 dpi. Again, St/CDA immunization was the strategy with the best performance showing the lowest parasitemia with a six-fold reduction of AUC compared with control (Figure 3B).

Considering the non-lethality of this model, weight loss was detected mainly in the chronic phase of infection (>100 dpi). While control mice lost about 30% of their body weight at the endpoint, vaccinated mice were able to maintain or even increase their weight during the course of infection (Figure 3C).

As *T. cruzi* infection progresses, tissue-associated damage might be presented in target organs. To characterize the protection levels achieved during the chronic phase, we analyzed multiple endpoints post-infection. At 120 dpi, electrical activity of heart was assessed by an electrocardiogram (ECG) determination. Age and sex-matched non-infected mice were incorporated in the analysis. Although not significant, except for St/CDA, all vaccinated and infected animals displayed a tendency to increase the PR interval in their ECG data, compared with non-infected mice (Figure 3D). A significant prolongation of the cQT interval was detected only in *T. cruzi*-infected Se/PBS groups compared to non-infected mice. Despite the fact that we did not detect differences between the immunized groups and infected controls, it should be noted that values recorded for all vaccinated animals were not significantly different from non-infected mice (Figure 3D). At 240 dpi, the activity levels of enzymes associated with tissue damage were determined in serum (Figures 3E,F). In agreement with ECG data, only *T. cruzi*-infected Se/PBS group presented higher levels of the cardiac isoform of creatine kinase (CK-MB) compared to non-infected animals. St-vaccinated mice showed a significant reduction of specific activity of CK-MB compared to Se/PBS (Figure 3E). Altogether, these results highlight the ability of St vaccination to ameliorate alterations of cardiac physiology during the chronic phase of *T. cruzi* infection.

As *T. cruzi* can also persist in skeletal muscle, serum CK activity was measured as a surrogate marker of tissue damage. All vaccinated mice displayed a clear reduction in CK levels, though higher levels of serum activity were detected in animals that received Sc/CDA formulation (Figure 3F).

To further characterize this scenario, parasitic load was analyzed by qPCR in target tissues, cardiac and skeletal muscle (Figure 3G). Preference of skeletal muscle persistence was detected in all animals, *T. cruzi*-DNA ratio _skeletalmuscle_/_heart_ = 4 (95% confidence interval: 2-6). St-immunized mice with CpG or CDA were able to reduce parasitic load in both target organs compared to the Se/PBS control group. In accordance with previous readouts, mice that received the Sc/CDA vaccine, in accordance with previous readouts, presented higher levels of parasite persistence, demonstrating a suboptimal control of the infection.

**Figure 4:**
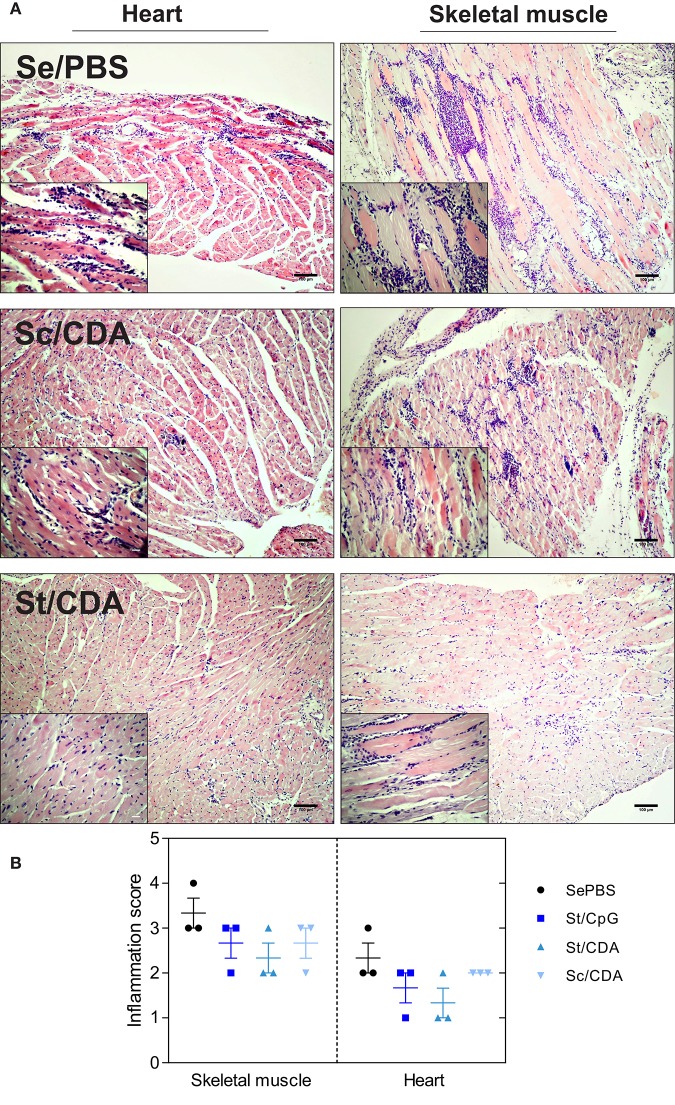
Histopathological evaluation of randomly sampled tissues from controls and vaccinated animals. Mice were vaccinated and infected with *T. cruzi* K-98 strain. At 240 dpi, histopathological analysis of *T. cruzi*-target organs was performed. **(A)** Representative tissue sections (H&E stained) for the indicated groups. Insets highlights mononuclear cell infiltrates in each tissue. Scale bar: 100 μm. **(B)** Graphs shows inflammation score semi-quantitatively evaluated for each group. Results are representative of two independent experiments.

Histology data indicated the presence of mononuclear cell infiltrates in both cardiac and skeletal muscle (Figure 4A). In correlation with parasite persistence, higher levels of chronic inflammation were observed in the latter (Figures 4A,B). Tissue sections from Se/PBS control mice presented signs of necrosis and chronic inflammation with multiple confluent inflammatory foci. On the other hand, St-immunized animals showed a decrease in the levels of mononuclear cell infiltrates while Sc/CDA presented the worst performance between immunized animals, with levels of inflammatory foci similar to control mice. These results highlight the inferiority of Sc/CDA vaccination in terms of parasite persistence, level of mononuclear cell infiltrate, and tissue damage compared to formulations bearing Traspain as anti-*T. cruzi* prophylactic vaccine.

### Antigen-Specific CD4^+^ T-Cell Response Differs in Functionality in St/CDA and Sc/CDA Groups

**Figure 5:**
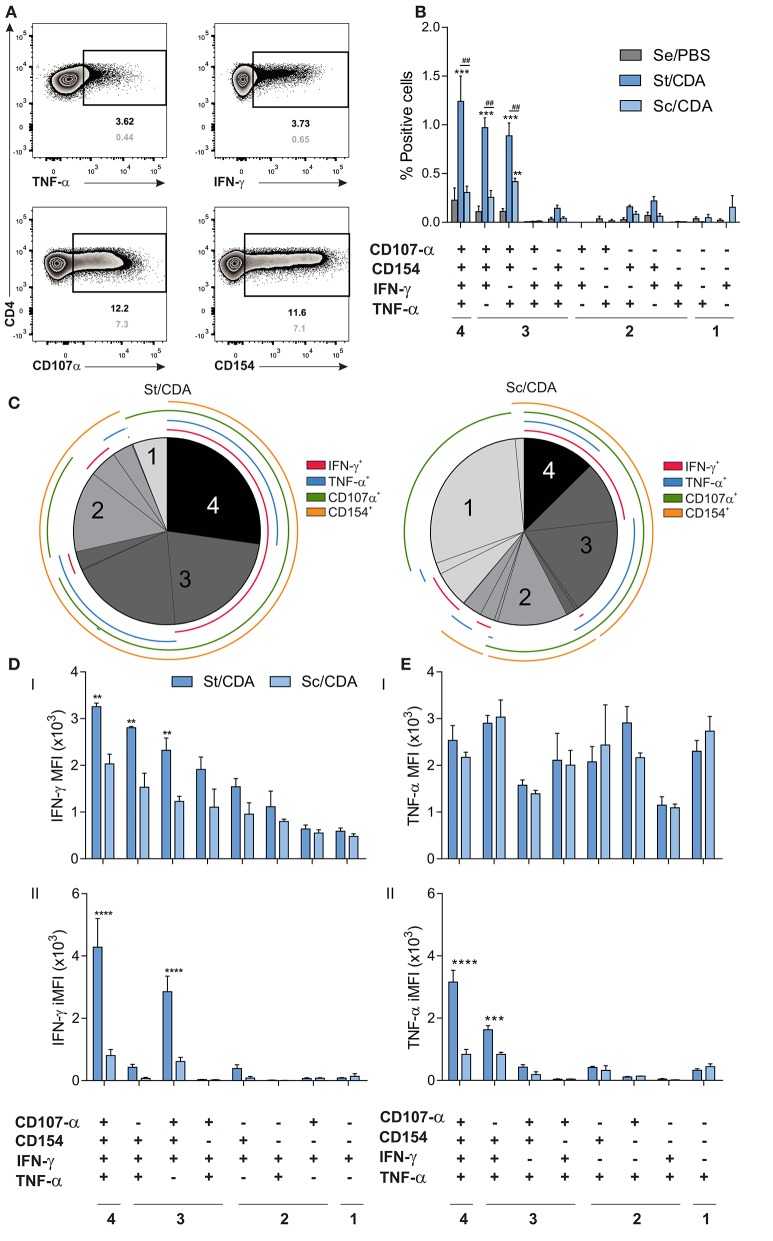
Polyfunctionality analysis of the CD4 T-cell compartment by flow cytometry. Female C3H mice were vaccinated as indicated, and 20 days post-immunization, spleen cells were restimulated *ex vivo* with Traspain. Surface and intracellular staining was performed, and Boolean combination gate strategy was carried out in order to assess simultaneous production of IFN-γ, TNF-α, CD154, and CD107α. **(A)** Representative Zebra plots showing the individual gates included in the Boolean combination strategy for the CD4 compartment. Frequency of positive events upon antigen re-stimulation or RPMI are shown in black and gray respectively. **(B)** Bar chart showing the frequency of cytokine producing subsets within CD4 T lymphocytes. Values were background subtracted. ***p < 0.001, **p < 0.01 comparing with Se/PBS, ^##^*p* < 0.01, between indicated groups, *n* = 3 mice per group, two-way ANOVA + Tukey's multiple comparisons test. **(C)** Pie chart showing fraction of the antigen-specific response for all positive subsets. Concentric lines are drawn to show the composition of each subset. Hierarchy of **(D)** IFN-γ and **(E)** TNF-α expression within functionally defined subsets of cytokine-producing cells. Both mean fluorescent intensity, MFI (I) and integrated MFI (II) are shown. ****p < 0.0001 ***p < 0.001, **p < 0.01 comparing between groups, two-way ANOVA + Sidak's multiple comparisons test. All results are representative of two independent experiments.

Considering the differences between groups that received Traspain or the combination of single antigens (Sc/CDA), we performed a FACS analysis of spleen cells to further analyze the quantity and quality of the cellular immune response triggered by each of these two formulations. To that end, mice were vaccinated, and flow cytometry was performed to assess all combinations of IFN-γ, TNF-α, CD154, and CD107α markers for the CD4^+^ T-cells subset by Boolean gating strategy upon antigen re-stimulation (Figure 5A).

A significant increase in the magnitude of multifunctional (4^+^ and 3^+^ functions) Traspain-specific population was detected in St/CDA group which showed a four-fold increase in the % 4^+^ cells compared to Sc/CDA (% of 4^+^ range from 1.6 to 0.8 and 0.4–0.2, respectively) (Figure 5B).

Boolean analysis revealed that while ~25% of Traspain-specific CD4 T cells expressed all four markers in St/CDA group, they only represent ~12% when mice received the Sc/CDA formulation (Figure 5C). On the contrary, single positive producers were highly represented in this group, where almost 40% of the antigen-specific response detected was monofunctional.

Higher IFN-γ MFI was detected in multifunctional cells in both groups. However, CD4 T cells from St/CDA group presented the highest levels in all subsets (Figure 5DI). Regarding TNF-α production, this scenario was not observed (Figure 5EI). Still, upon calculation of the integrated MFI (iMFI), a metric that encompass the magnitude (frequency) and the quality (fluorescence intensity) of this cytokine, the same trend was detected (Figure 5EII). Similarly, a marked contrast was observed in the iMFI of IFN-γ production between both groups, a fact that highlights the better sensibility of this measurement for comparing functionality among different formulations (Figures 5DII**, E**II). Altogether, these results point to a higher quality of helper T lymphocytes primed by the St/CDA formulation that might contribute to efficacy differences observed.

### CD8^+^ T-Cell Functionality Is Improved in St/CDA Group

**Figure 6:**
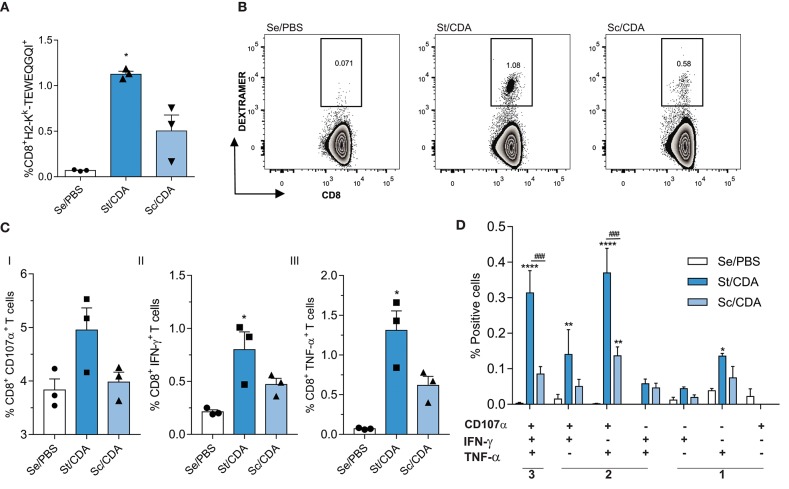
Enhancement of CD8^+^ T-cell-mediated immune responses in Traspain-vaccinated female C3H mice. **(A)** Priming of pathogen-specific CD8+ T cells by vaccination at 30 dpi. **(B)** Representative dot-plots for the indicated groups. Spleen cells were restimulated *ex vivo* with TEWETGQI peptide. After cell staining, Boolean gate strategy was performed in order to assess simultaneous production of CD107-α, IFN-γ, and TNF-α **(C)** total frequency of CD8 T cells producing each marker. Values were background-corrected, *p < 0.05 against control group (Se/PBS), one-way ANOVA Kruskal-Wallis test + Dunn's multiple comparisons test. **(D)** Frequency of cells expressing each of the seven possible combinations of cytokines. ****p < 0.0001, **p < 0.01 comparing with Se/PBS, ^###^*p* < 0.001 between the indicated groups, two-way ANOVA + Tukey's multiple comparisons test. Results are representative of two independent experiments.

As CD8^+^ T cells play a pivotal role in controlling *T. cruzi* infection, we analyzed the priming of pathogen-specific cells by surface staining, employing an MHC-I dextramer loaded with the peptide TEWETGQI, an immunodominant peptide from the ASP2 region of Traspain (Figure 1A). We observed an expansion of antigen-specific CD8^+^ T cells in both vaccinated groups. However, mean values were significantly higher only in St/CDA group compared to controls (Figure 6A). A two-fold increase in its peptide-specific CTL frequency was observed when we compared individuals from both treated groups revealing a more robust CTL response in this group. A similar trend between groups was observed when production of IFN-γ, TNF-α, and CD107α was analyzed (Figures 6CI–III).

**Figure 7:**
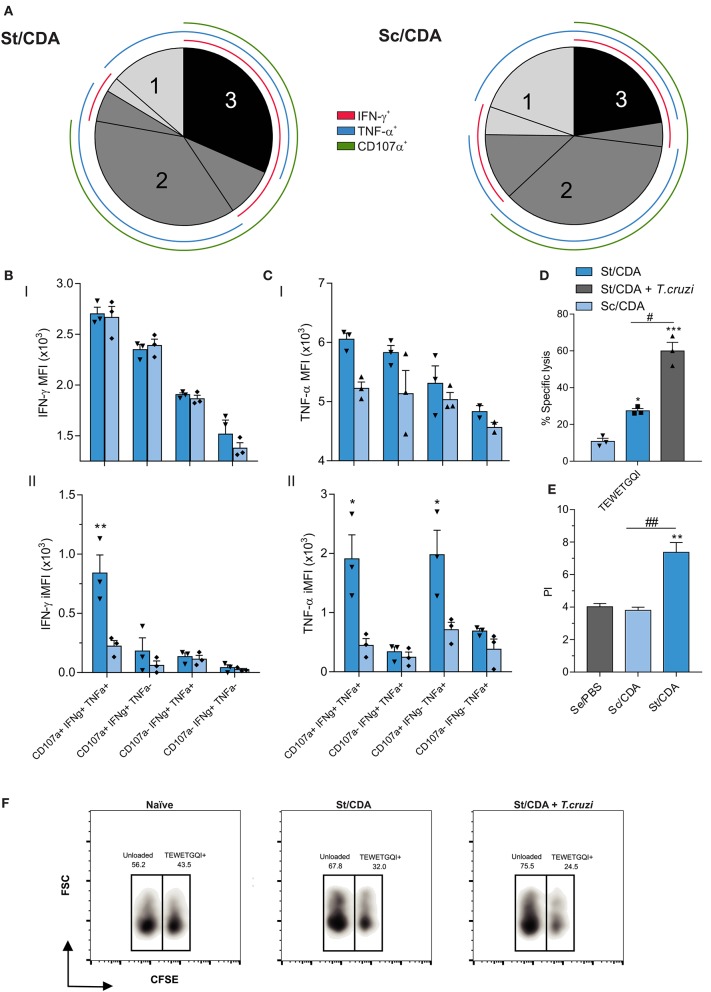
Polyfunctionality analysis of CD8 T-cell compartment by flow cytometry. **(A)** Pie chart showing the percentage of each functional subset from the total antigen-specific response for female C3H vaccinated animals. Color coded concentric lines indicate each cytokine or marker. Hierarchy of **(B)** IFN-γ and **(C)** TNF-α expression within functionally defined subsets of responding cells. Both mean fluorescent intensity MFI (I) and integrated MFI (II) are shown. *p < 0.05 **p < 0.01 comparing between groups, two-way ANOVA + Sidak's multiple comparisons test. **(D)** *in vivo* CTL assay. Spleen cells from female C3H donor mice were loaded with TEWETGQI peptide or unloaded. Cells were stained with CFSE, and intravenously injected to syngeneic naïve, vaccinated (St/CDA, Sc/CDA) or St/CDA-vaccinated and *T. cruzi* RA infected mice at 45 dpi. *p < 0.05 ***p < 0.001 against Sc/CDA group. ^#^*p* < 0.05, between the indicated groups, one-way ANOVA + Tukey's multiple comparisons test. Results are expressed as mean ± SEM, and represent at least three independent experiments, *n* = 3 per group. **(E)** 3H-Thymidine incorporation assay. Spleen cells from *T. cruzi*-RA-infected female mice were removed at 100 dpi and recalled with 20 μg/ml of F105 *T. cruzi* lysate. Results are expressed as proliferation index (PI). ^##^*p* < 0.01 between indicated groups. **p < 0.01 comparing with Se/PBS. one-way ANOVA + Tukey's multiple comparisons test, *n* = 6 per group. **(F)** Representative density-plot of *in vivo* CTL assay showing percentage of CFSE populations in the indicated groups. All results are representative of two to three independent experiments.

Employing Boolean strategy, we assessed all combinations of these three markers. St/CDA showed an increase in the frequency of marker combinations compared to Sc/CDA, specifically a four-fold increment was observed in the magnitude of 3^+^ CD8 T cells (Figure 6D). Taking into account the total antigen-specific CD8 response detected, this multifunctional CTL subset represents 32% in St/CDA group vs. 22% in Sc/CDA (Figure 7A).

Differences in the production levels of cytokines between groups were observed in TNFα but not in IFNγ-producing subsets as MFI analysis revealed (Figures 7B,CI). However, considering the frequency of each subpopulation, mice vaccinated with St/CDA displayed a clear difference in both cytokine subsets (Figures 7B,CII). Consequently, a significant four-fold increase in the IFNγ-iMFI of 3^+^ polyfunctional subset was detected when we compared both formulations.

In order to confirm the cytotoxic potential of CD8^+^ T lymphocytes generated by St/CDA formulation, we performed an *in vivo* cytotoxicity assay where we transferred splenocytes loaded with TEWETGQI peptide from a syngeneic donor to naïve, immunized, or immunized and infected mice (Figure 7D). Hence, St/CDA vaccinated animals presented around 30% lysis of TEWETGQI^+^ cells. Nearly half of this value was observed in Sc/CDA. The ability of the clone to re-expand was confirmed by its increased cytotoxic activity upon *T. cruzi* infection, reaching values of about 60% lysis in immunized mice at 45 dpi and further demonstrating that the functionality of this subset is still preserved after parasite infection (Figures 7D,F). In a similar fashion, higher proliferation potential was detected in spleen cells from St/CDA at 100 dpi upon antigen-specific re-stimulation (Figure 7E). As T-cell response plays a key role for the elimination of infected cells in *T. cruzi* target tissues, its functionality represents an essential feature for the immune mediated control of *T. cruzi* infection induced by prophylactic vaccination.

## Discussion

The definition of a correlate of protection for anti-*T. cruzi* vaccines is still missing. This fact is related with the complexity of the immune response required to control parasite invasion and intracellular replication.

We have recently introduced Traspain, a unique chimeric antigen based on key *T. cruzi* antigens that proved to be effective for the control of experimental infection in a subunit vaccine model employing CDA as an adjuvant. The results shown here indicate that novel heterologous prime-boost strategies should be focused on obtaining robust polyfunctional T-cell responses in order to be an effective regimen to trigger anti-parasitic cell-mediated immunity.

The profile of the immune response triggered was influenced not only by the adjuvant employed in boost doses but also by the antigen in each regimen. In agreement with previous results (10), mice that received CDA in boost doses displayed a Th1/Th17 bias with a balanced cytokine profile. In contrast, a prime-boost regimen designed to trigger TLR9 employing CpG as boost adjuvant showed an immune response consistent with a Th1 polarized profile (Figure 1F). The robustness of the immune response obtained with these two adjuvants was different in terms of IL-17, IFN-γ, and IL-4 secretion. In addition to the differences in the signaling pathway, this fact might be related to several variables that we cannot rule out like dissimilarities in TLR9 and STING distribution in mice nasal mucosa, differences in the stability of these two small molecules, and the state of the immune system after oral DNA priming by live-attenuated bacteria. All these might contribute to a more efficient boost potency of CDA over CpG.

Interestingly, the single antigens combined in the Sc/CDA group, failed to achieve similar levels of vaccine potency despite receiving CDA. This fact clearly emphasizes the importance of the immunogen, as we have previously observed a similar scenario in the subunit vaccine model where Traspain showed an improved priming efficiency compared to the formulation and administration of the main two domains alone (10). Even though the difference between CMI can be attributed to the presence of iTS linker in the Traspain formulation, the likelihood of this scenario seems to be on the low side considering the short length of the sequence (only 25 amino acids) and the lack of known immunodominant epitopes in that region of the molecule. We have previously determined that the linker region from iTS can be targeted by antibodies and CTL response in Traspain/CDA vaccinated mice.

The more balanced and robust immune response triggered by a CDA boost within the same immunogen highlights its advantageous use for mucosal prime-boost strategies over other adjuvants or its inclusion in the design of novel adjuvant systems, an strategy that has been proved to have a positive effect on immunogenicity and efficacy against intracellular pathogens (47).

In terms of vaccine efficacy, we demonstrated that there was a clear correlation with immunogenicity, since St/CDA immunized mice showed an enhanced immune response that was then associated with a reduction of circulating parasites and an increase in survival rates upon a lethal *T. cruzi* challenge (Figure 2).

Considering that vaccine efficacy can be higher against vaccine-like strains compared to others from a different genetic background, infection with a parasite from DTU I, clone K-98 was tested. This *T. cruzi* clone has several useful characteristics. It has a low virulence in the murine model, a slow replication rate, and, given its DTU I background, is likely to have a more discrete antigenic repertoire compared to DTU VI strains like RA, employed here for lethal assays (48). Importantly, given its non-lethality, this infection model allows us to evaluate vaccine performance throughout a longer infection time without reducing the initial load of parasite inoculum.

Regarding vaccine efficacy, we found a similar profile in both acute and chronic infection models, St/CDA immunization being the one that produced the strongest reduction of blood parasites and an important reduction of body weight loss compared with the non-vaccinated control.

As cardiac muscle is one of *T. cruzi*'s target tissues, murine ECG was employed as a tool for assessing cardiac physiology. Its data revealed an improved outcome on vaccinated animals, which suffered fewer alterations (QTc and PR interval prolongation), compared to Se/PBS infected mice. Similar alterations were previously observed in other *T. cruzi*-infected mice (6, 49). Interestingly, between vaccinated groups, Sc/CDA presented the worst performance. This group, like infected controls, showed higher alterations of ECG at 120 dpi, a fact that was then associated with increased CK-MB serum activity, parasite persistence, and mononuclear cell infiltrate at endpoint at both the cardiac and skeletal muscle level. These readouts indicate a suboptimal control of the disease progression in these animals. Even though cardiac damage was detected by CK-MB activity in sera at endpoint, parasite persistence determined by qPCR, was higher in skeletal muscle, representing one shortcoming of the infection model.

Beneficial effect of IL-17 might be related with the improvement in the outcome of St/CDA vaccinated group compared to St/CpG. Presence of IL-17 secreting cells might contribute to an improved priming of CTL responses (30) and to the control of an otherwise extreme pro-inflammatory immune response (50). The latter is a common scenario of lethal acute *T. cruzi* infections and may explain differences in protection observed upon RA challenge, a similar situation was observed with hypervirulent strains of *M. tuberculosis* (23). However, in chronic infection models like the K-98 clone-male, C3H mice we were not able to detect clear-cut differences between each formulation, at least in the analyzed readouts. Considering that Th17 cells play a role in autoimmune diseases that are associated with chronic inflammation, the lack of an overwhelming inflammatory response in vaccinated animals suggests that the Th17 cells primed did not undergo a maturation process that would have led them to acquire a pathogenicity state upon *T. cruzi* infection.

Given that St/CDA and Sc/CDA groups performed so distinctly and considering the differences previously observed in the subunit vaccine model (10), we speculate that priming of CD4 and CD8 T cells might be compromised in the latter. To test this hypothesis, we further analyzed the quantity and quality of the cell-mediated immunity triggered by these two formulations by flow cytometry. Indeed, Boolean gating strategy revealed key functional differences between each other in both antigen-specific CD4 and CD8 T-cell compartments (Figures 5–7).

Higher levels of poly-functional T-cell subsets has been directly related with an improved efficacy in other vaccine models against intracellular pathogens (51, 52). In agreement with this observation, an improved outcome was detected in the St/CDA group, which showed an increase in the frequency of poly-functional CD4 and CD8 T-cell subsets.

Interestingly, bigger differences were observed at the CD4 compartment, where more than 70% of the antigen-specific response produced 3^+^ and 4^+^ functions. This fact might be influenced by the stimulation protocol since the CD4 compartment was stimulated by whole recombinant Traspain, while the CD8 compartment was stimulated only with TEWETGQI peptide. Employing a peptide pool for recalling T cells can solve this issue in upcoming studies. On the other hand, increasing the sample size would let us detect differences among groups that might be underestimated in this study.

Another observation that might contribute to the improved control of *T. cruzi* progression in St/CDA groups might be associated with the higher quality of multifunctional subsets as was demonstrated by its higher ability to produce more of each cytokine compared to less functional ones as well as higher *in vivo* CTL activity. This scenario was also observed with other vaccines against parasitic disease such as leishmaniasis (51) and malaria (53).

We believe that multifunctional T-cell priming would be a desirable attribute for a T-cell-based vaccine against *T. cruzi* considering that it involves not only a higher effector function but also a greater long-term memory potential, as single positive cells are associated with terminal effector T lymphocytes (54). Interestingly, monofunctional responses have been observed in *T. cruzi* chronically infected patients (55) and a higher functionality has been observed in patients with less severe forms of chronic Chagas cardiomyopathy (24, 56), as well as in *T. cruzi*-infected children (55), a stage of life where parasitic cure by drug treatment is possible.

In the murine model, the functionality of CTL responses during the chronic phase is essential for controlling parasite burden (57), and improving it through active immunotherapy or drug combination therapy, though challenging, seems an attractive area of research. Even though heterologous prime-boost immunization represents an interesting strategy to optimize T-cell responses, we showed that fine-tuning is also possible not only by varying the nature of the adjuvant employed in the subunit vaccine type but also by changing the nature of the antigen. Therefore, constructing new molecules in order to improve immunogenicity should be further studied. The results presented here reinforce the notion that measurement of T-cell polyfunctionality is a key factor that needs to be considered in the definition of a correlate of protection for the design of novel anti-parasitic vaccines and that the analysis of T-cell responses against protective parasitic antigens for the rational design of novel anti-*T cruzi* vaccines should be further extended.

## Data Availability Statement

All datasets generated for this study are included in the article/supplementary material.

## Ethics Statement

This animal studies were reviewed and approved by the Review Board of Ethics of the School of Medicine, UBA, Argentina (Resol. C.D. #3721/2014) following the guidelines established by the National Research Council, and conducted in accordance to the regulations of Lower Saxony No. 09.42502 04 105/07, Germany. Animal sample size was estimated by a power-based method.

## Author Contributions

AS, AB, SC, and EM designed experiments. AS, AB, MM, NC, KS, SW, TE, GG, CM, AC, and SC performed experiments. AS, AB, and KS analyzed data. AS, AB, NC, SC, CG, and EM discussed data. AS and EM wrote the manuscript. AS, CG, and EM revised the manuscript.

### Conflict of Interest

The authors declare that the research was conducted in the absence of any commercial or financial relationships that could be construed as a potential conflict of interest.
